# Time-series lipidomic analysis of the oleaginous green microalga species *Ettlia oleoabundans* under nutrient stress

**DOI:** 10.1186/s13068-018-1026-y

**Published:** 2018-02-06

**Authors:** E. K. Matich, M. Ghafari, E. Camgoz, E. Caliskan, B. A. Pfeifer, B. Z. Haznedaroglu, G. E. Atilla-Gokcumen

**Affiliations:** 1 Department of Chemistry, University at Buffalo, The State University of New York (SUNY), Buffalo, NY 14260 USA; 2 Department of Civil, Structural and Environmental Engineering, University at Buffalo, The State University of New York (SUNY), Buffalo, NY 14260 USA; 3 Department of Chemical and Biological Engineering, Koc University, 34450 Istanbul, Turkey; 4 Institute of Environmental Sciences, Bogazici University, 34342 Istanbul, Turkey; 5 Department of Chemical and Biological Engineering, University at Buffalo, The State University of New York (SUNY), Buffalo, NY 14260 USA

**Keywords:** Lipidomics, Microalgae, Biochemicals, Metabolomics

## Abstract

**Background:**

Microalgae are uniquely advantageous organisms cultured and harvested for several value-added biochemicals. A majority of these compounds are lipid-based, such as triacylglycerols (TAGs), which can be used for biofuel production, and their accumulation is most affected under nutrient stress conditions. As such, the balance between cellular homeostasis and lipid metabolism becomes more intricate to achieve efficiency in bioproduct synthesis. Lipidomics studies in microalgae are of great importance as biochemical diversity also plays a major role in lipid regulation among oleaginous species.

**Methods:**

The aim of this study was to analyze time-series changes in lipid families produced by microalga under different nutrient conditions and growth phases to gain comprehensive information at the cellular level. For this purpose, we worked with a highly adaptable, oleaginous, non-model green microalga species, *Ettlia oleoabundans* (a.k.a. *Neochloris oleoabundans*). Using a mass spectrometry-based untargeted and targeted metabolomics’ approach, we analyzed the changes in major lipid families under both replete and deplete nitrogen and phosphorus conditions at four different time points covering exponential and stationary growth phases.

**Results:**

Comprehensive analysis of the lipid metabolism highlighted the accumulation of TAGs, which can be utilized for the production of biodiesel via transesterification, and depletion of chlorophylls and certain structural lipids required for photosynthesis, under nutrient deprived conditions. We also found a correlation between the depletion of digalactosyldiacylglycerols (DGDGs) and sulfoquinovosyldiacylglycerols (SQDGs) under nutrient deprivation.

**Conclusions:**

High accumulation of TAGs under nutrient limitation as well as a depletion of other lipids of interest such as phosphatidylglycerols (PGs), DGDGs, SQDGs, and chlorophylls seem to be interconnected and related to the microalgal photosynthetic efficiency. Overall, our results provided key biochemical information on the lipid regulation and physiology of a non-model green microalga, along with optimization potential for biodiesel and other value-added product synthesis.

**Electronic supplementary material:**

The online version of this article (10.1186/s13068-018-1026-y) contains supplementary material, which is available to authorized users.

## Background

Microalgae species are valuable biomass resources for the advancement of commercial applications in biofuel, nutraceutical, pharmaceutical, and environmental sectors [1–3]. High efficiency in photosynthesis, rapid growth in minimized land, and the capability to utilize recycled inputs such as carbon dioxide, nitrogen, and phosphorus from waste streams contribute to sustainability metrics and offer economic feasibility for large-scale commercialization [4, 5]. One of the key aspects of biotechnological exploitation in microalgae focuses on lipids [6, 7]. In particular, green microalgae species accumulating lipids as energy storage molecules under stress conditions have gathered varied interests for potential applications [5, 8, 9]. Key examples include essential fatty acids (FAs) for nutraceuticals [10, 11], triacylglycerols (TAGs) for biodiesel applications [6, 12–14], and carotenoids for nutraceuticals and food additives [15–17].

From a cellular point of view, lipids are not only building blocks for value-added biochemicals but also structural components of cellular membranes and plastids [18–20]. Though not comprehensively explored in algae, lipids and their metabolites also take part in signaling pathways [20–22]. Consequently, understanding lipid trafficking and compartmentalization in microalgae represents an opportunity for improved understanding of cellular functionality and improved biomass yields towards final product optimization.

From a metabolic point view, a majority of green microalgae, generally referred to as oleaginous species, accumulate more lipids under nutrient stress conditions [23–25]. However, the lack of a macronutrient adversely affects cellular homeostasis and, thus, cellular growth stagnates. This is a major topic of interest for algal scientists and researchers investigating optimal conditions leading to higher lipid yields and sustained cellular growth profiles.

Studies in model species such as *Chlamydomonas reinhardtii*, *Chlorella* sp., and *Nannochloropsis* sp. [26–28] have identified key mechanisms of lipid metabolism in green microalgae. Meanwhile, new sets of questions emerged with *Nannochloropsis* which differs in lipid metabolism among other microalgae species [27, 29]. This was also evident in other species as the regulation of multi-sub-unit enzyme complexes, such as the acetyl-CoA carboxylase and alcohol dehydrogenase, with roles in fatty acid metabolism differ in response to varying stress conditions [30, 31]. Trafficking of free FAs between chloroplast, cytosol, and endoplasmic reticulum also shows dissimilarity, resulting in an uneven accumulation of lipid bodies that mainly consist of TAGs, important products for biodiesel production, among microalgae species [27, 32–34].

To resolve lipid regulation and metabolic fractionation of lipids in microalgae, high-resolution mass spectrometry (HR-MS) lipidomic studies are required. HR-MS is advantageous in providing information at the molecular level in addition to being utilized in a high-throughput manner to study the composition of complex extracts. In this study, we utilized a liquid chromatography–quadrupole time-of-flight mass spectrometry (LC–QToF MS) based approach developed by our group to analyze differences in lipid composition in the non-model microalga species *Ettlia oleoabundans*, formerly known as *Neochloris oleoabundans* [35]. The reason for choosing *E. oleoabundans* as the target species is its native propensity for lipid accumulation up to 35–54% of its dry cell weight (DCW) without any genetic modification [36, 37]. Due to high quality and quantity of neutral lipids [38], *E. oleoabundans* is considered as an oleaginous species and a viable candidate for the commercial exploration towards production of biofuels and other value-added products [39, 40].

Here, we investigated the effects of nitrogen and phosphorus deplete conditions (N− and P−) compared to replete conditions (N+ and P+) in a time-series manner, covering early and late exponential and stationary phases. Combining a targeted and untargeted metabolomics approach with temporal variation in cellular growth, we were able to gain detailed and comprehensive information on the biochemical changes that take place in *E. oleoabundans* over time and under nutrient stress. This level of detailed characterization both at the temporal and metabolite coverage level in this organism, to the best of our knowledge, has not been carried out before.

## Methods

### Materials

Liquid chromatography - mass spectrometry (LC–MS) grade methanol and high-performance liquid chromatography (HPLC) grade isopropanol were purchased from Millipore Sigma (Billerica, MA). Formic acid, ammonium formate, and ammonium hydroxide were purchased from Sigma-Aldrich (St. Louis, MO). HPLC grade chloroform was purchased from Honeywell Burdick & Jackson (Muskegon, MI). Glass beads (0.5 mm) were purchased from Bertin Technologies (Montigny, France). Stock solutions of lipid standards, purchased from Avanti (Alabaster, AL), were prepared in chloroform and kept at − 80 °C until analysis. The columns for LC–MS analysis were purchased from Phenomenex (Torrance, CA) and included a C5 Luna column (50 × 4.6 mm, 5 μm) and a C18 Gemini column (50 × 4.6 mm, 5 μm) for electrospray ionization positive (ESI+) and negative (ESI−) modes. Nanopure water was used for all analyses (resistivity 18.2 MΩ-cm at 25 °C).

### Experimental methods

#### Microalgal strain, media, and cultivation conditions

*Ettlia oleoabundans* (strain UTEX1185 from the culture collection of University of Texas at Austin) was grown under nitrogen replete (N+) ([NO_3_^−^] = 7.14 mM), nitrogen deplete (N−) ([NO_3_^−^] = 0.714 mM), phosphorus replete (P+) ([PO_4_^3−^] = 1.808 mM), and phosphorus deplete (P−) ([PO_4_^3−^] = 0.064 mM) conditions. Microalga was grown in modified Bold 3N growth medium in 500 mL photobioreactors (PBRs) for 11 days. Five biological replicates were used for each condition. PBRs were used to grow algae at 25 ± 2 °C, under a light:dark cycle of 14:10 h using fluorescent light (32W Ecolux, General Electric, Fairfield, CT) and there was a 110 μmol-photon/m^2^/s photosynthetic photon flux density. A flow rate of 200 mL/min using a mass flow controller (Cole-Parmer Instrument Company, IL) was used to aerate the PBRs continuously using sterile, activated carbon-filtered air without additional CO_2_.

#### Determination of optical density, nitrate, and phosphate concentrations in the media, dry biomass, and lipid composition

The optical density and nitrate and phosphate concentrations in the media were analyzed each day, while dry biomass and lipid composition were analyzed at time points of interest, i.e., days 2, 4, 7, and 10, corresponding to early exponential, late exponential, early stationary, and late stationary phases, respectively. The optical density was measured at 730 nm using a microwell-plate reader (SpectraMax i3, Molecular Devices, CA). The total nitrate and phosphate concentrations in culture media were determined daily by a Microplate Nitrate Kit (NECi, Lake Linden, MI) and a Phosphate Colorimetric Assay Kit (BioVision, Milpitas, CA). The dry biomass was analyzed by filtering 10 mL of the microalga growth suspension using vacuum filtration and drying at 105 °C for 2 h. The dry biomass values are reported in Additional file 1: Table S1.

The lipid composition was calculated by extracting the lipid content of cells by taking 45 mL of each culture, centrifuging at 2934×*g*, and resuspending the pellets in 1 mL of methanol. The suspension was homogenized by bead-milling in a vial containing 0.1 and 0.5 mm diameter glass beads. The suspension was then transferred to a new glass vial. Next, 5 mL of 2-ethoxyethanol was mixed with the methanol solution and the vial shaken (200 rpm) at 60 °C for 30 min. Solid residues were removed by passage through 1.2 μm glass microfiber filters. Later, the solvent was evaporated, and the extract was weighed and resuspended in 3 mL of 2:2:1:1 hexane:toluene:acetone:methanol mixture to remove extracted proteins and other non-lipid compounds. Finally, the solution was decanted, and individual vials were dried at 60 °C and re-weighed. The percent lipid composition was calculated as (precipitated lipid weight/total dry weight) * 100.

#### Metabolite extraction and biomass normalization

The hydrophobic metabolites were extracted following a procedure described in detail previously [35]. Briefly, pellets were resuspended in methanol spiked with C18:1 fatty acid-*d*9 (henceforth referred to as oleic acid-*d*9) and disrupted by bead-milling. The resulting solution was mixed with chloroform and water (resulting in a mixture of 2:1:1 chloroform:methanol:water) for three serial biphasic extractions. The chloroform layers from three extractions were combined. During the extraction, the samples were spiked with TAG (19:0/19:0/19:0) to serve as an additional surrogate to calculate the extraction efficiencies. The combined chloroform layers were then rotary evaporated and resuspended in chloroform. The volume of chloroform used to resuspend these samples varied based on the differences in biomass, such that each sample had approximately 9.20 mg of biomass/mL.

#### Extraction efficiency calculations

The extraction efficiencies of oleic acid-*d*9 and TAG (19:0/19:0/19:0) were calculated using matrix-matched calibration curves. The abundances of the internal standards in each sample were then used to calculate concentrations based on the matrix-matched calibration curve. The percent recovery was calculated as [measured concentration of surrogate in sample spiked before the extraction (µM)/theoretical concentration (µM)] * 100.

#### LC–QToF-MS-based data acquisition

The normalized extracts were analyzed using an Agilent 1260 HPLC unit (equipped with a degasser, binary pump, autosampler, temperature gauge, and isopump) that was coupled to an Agilent 6530 Quadrupole Time-of-Flight (QToF) mass spectrometer (MS) with a Dual Jet Stream electrospray ionization (ESI) source. Two independent profiling experiments with three biological replicates (*n* = 3) from 16 growth conditions (N−, N+, P−, and P+ conditions each sampled individually on days 2, 4, 7, and 10) were analyzed both in ESI+ and ESI− ionization modes. The samples were run in a randomized order to prevent any run-dependent biases. Mobile phase A was composed of 95:5 water/methanol mixture and mobile phase B was made of 60:35:5 isopropanol/methanol/water, supplemented with either 0.1% ammonium hydroxide for ESI− or 0.1% formic acid and ammonium formate for ESI+. The starting flow rate was 0.1 mL/min of 100% A, which was then increased to 0.5 mL/min after 5 min; the mobile phase composition was changed gradually from 100% A to 100% B from 5 to 45 min, and was maintained at 100% B for 10 min. The mobile phase was then equilibrated back to 100% A and kept there for 8 min at 0.5 mL/min. The total run length was 63 min. For analysis under ESI+, a C5 column was used for chromatographic separation to allow the analysis of highly hydrophobic biodiesel precursor lipids, TAGs, while a C18 column was used for analysis under ESI−. The data acquisition was carried out in extended dynamic range mode and *m*/*z*’s between 250 and 1700 were analyzed.

#### Untargeted analysis and LC–MS/MS identification

The resulting raw data were imported into MassHunter Profinder (version B.06.00, Agilent Technologies) for peak alignment and extraction of features. The recursive feature extraction was used to extract and align molecular features in all samples. This was intended for the deconvolution of chromatograms and alignment of molecular features across all sample data files according to mass and retention time. The parameters for the molecular feature extraction included: a peak height of ≥ 300 ion counts and possible ions including [M+H]^+^, [M+Na]^+^, [M+NH_4_]^+^, and [M−H]^−^. The alignment also involved isotope grouping restrictions including: a peak spacing tolerance of 0.0025 *m*/*z* and 7.0 ppm and a maximum charge state of 2. It was also required that there were at least two or more ions for a single molecular feature. In addition, for binning and alignment purposes, a tolerance of 0.3 min for a retention time window was set along with a mass window of 20 ppm. Some of the post-processing filters included: an absolute height filter of ≥ 3000 ion counts and the requirement for the molecular feature to be present in at least two out of three replicates in one experimental group. There was also a restriction set for the extracted ion chromatograms of an expected retention time range of ± 1.5 min, along with an acceptable data variation (across samples) of 5.6 ppm for the *m*/*z*_observed_ and 0.2 min for the retention time for each molecular feature.

The resulting aligned features were analyzed in Mass Profiler Professional (MPP, version B12.6.1 Agilent Technologies) for statistically significant differentiations between sampling conditions. Briefly, in MPP, the features were first filtered by frequency (> 66% or two out of three replicates for at least one sample group). Next, for statistical significance, analysis of variance (ANOVA) at 95% confidence level with a post hoc Tukey honest significant difference test and Benjamini–Hochberg false discovery rate (FDR) correction was performed. This process was carried out for two independent profiling experiments and the resulting feature lists were compared and the common features were kept. These *m*/*z*’s were then manually inspected for mass accuracy and abundance in Qualitative Analysis (version B.06.00 Agilent Technologies), and reintegrated if necessary. The retention time shifts observed between the first and the last sample injected were around 0.5 min. This list was then analyzed using a more stringent filtering and statistical criteria (a frequency of 100% and a *p* value of < 0.01). The resulting *m*/*z*’s were searched in METLIN [41] and Lipid Maps [42] databases to determine one or more possible candidate metabolites based on accurate mass and adduct. Known lipid standards (or lipids belonging to the same lipid families) were purchased for the candidate lipids. The liquid chromatography–tandem mass spectrometry (LC–MS/MS) data were collected similar to the descriptions provided in “LC–QToF-MS-based data acquisition” section, except fragments were observed at collision energies of 15, 35, 55, and 75 eV. The tandem mass spectrometry (MS/MS) fragmentation patterns of the *m*/*z* of interest and similar standards were compared. Searches based on MS/MS fragments provided in METLIN were also used in addition to the fragmentation patterns of known standards.

#### Targeted analysis of endogenous lipids

Targeted analysis of *m*/*z*’s from major lipid families was carried out by extracting the *m*/*z*’s of the most abundant adduct for each lipid family.

## Results and discussion

### Growth curves, lipid composition, and nutrient concentrations in the media

**Fig. 1:**
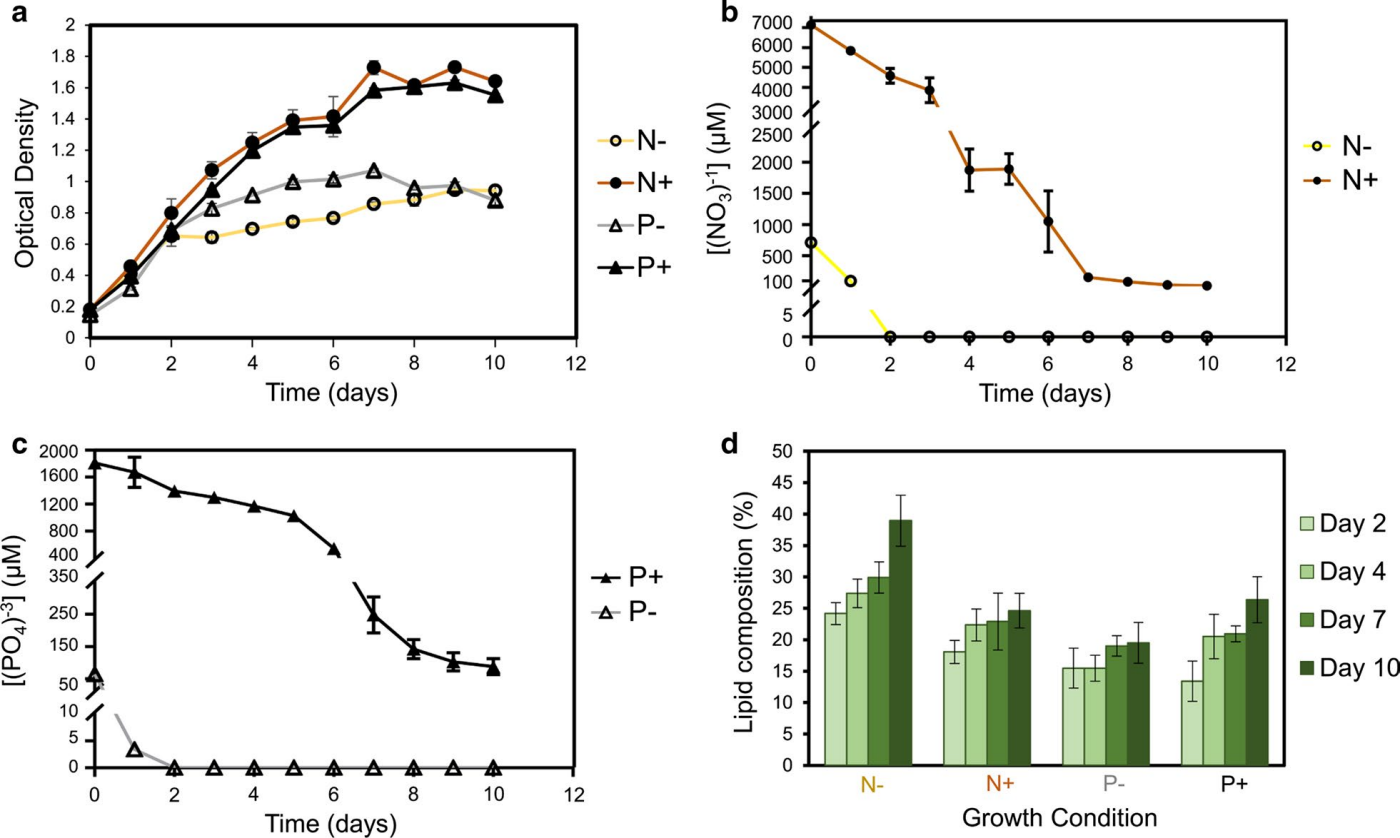
Growth rate, nitrate and phosphate concentrations in the media, and lipid composition. **a** Growth rate measured by optical density (at 730 nm). **b** Nitrate concentrations (µM) in media over time. **c** Phosphate concentrations (µM) in media over time. **d** Lipid composition ([lipid biomass/total biomass] * 100) of the microalga cells at different time points

*Ettlia oleoabundans* cells were grown under four different nutrient conditions (N−, N+, P−, and P+) and sampled at days 2, 4, 7, and 10, representing early and late exponential and early and late stationary growth phases, respectively. This matrix allowed insights on temporal changes in the lipid composition during different growth periods and under varying nutrient conditions. Five biological replicates from different PBRs were analyzed for optical density (OD) (Fig. 1a), remaining nitrate ([NO_3_^−^]) and phosphate ([PO_4_^3−^]) concentrations in the growth media (Fig. 1b, c, respectively), and total lipid composition (Fig. 1d).

Based on the growth curves monitored daily, early exponential (day 2), late exponential (day 4), early stationary (day 7), and late stationary (day 10) growth phases were confirmed (Fig. 1a). Total nitrate and phosphate in growth media were internalized completely by day 2, confirming the initiation of nutrient-depleted conditions in N− and P− reactors; meanwhile, residual nitrate and phosphate remaining in N+ and P+ reactors sustained nutrient replete conditions (Fig. 1b, c). As microalgal cells continued to utilize already internalized nutrients, biomass growth continued until early stationary phase at day 7 (Fig. 1a).

We measured total lipid contents at respective growth phases to assess the impact of varying nutrient conditions on overall biomass composition (Fig. 1d). Changes in the accumulation of total lipids were not statistically significant between P− vs. P+ except day 2 in P+ reactors. However, we observed significant increases over time in total lipid contents under N− conditions compared to N+ (Fig. 1d). In general, more lipids accumulated over time, though P− did not result in significant increases in lipid content of *E. oleoabundans.* In contrast, P+ reactors showed slightly higher levels of lipids. This was contrary to increased levels of lipid content observed in *Chlorella* sp. exposed to phosphorus limitation [43]. Xin et al. also observed decreased lipid content with respect to increased phosphorus concentrations in *Scenedesmus* sp. LX1 [44]. However, there are also reported studies of phosphorus limitation causing decreased lipid content in *Nannochloris atomus* and *Tetraselmis* sp. [45]. Meanwhile, N− had a more profound effect similar to the observations of most other green microalgae species accumulating more lipids under N− (Fig. 1d) [7, 46, 47]. A comparison of lipid accumulation in different green microalgae species exposed to similar nutrient variations is provided in Additional file 1: Table S2. Comparing the changes in different lipid classes, we observe a few major differences between *E. oleoabundans* and other green microalgae species such as *C. reinhardtii*, *Chlorella* sp., and *Nannochloropsis* sp. First, monogalactosyldiacylglycerols (MGDGs), which are one of the most abundant plant lipids in green microalgae [48], are depleted under N− in *C. reinhardtii*, *Chlorella* sp., and *Nannochloropsis* sp. However, these lipids are present at very low levels in *E. oleoabundans* in the current growth conditions (i.e., only C34:4- and C34:6-MGDGs are detected). Monogalactosylmonoacylglycerols (MGMGs) and phosphatidic acids (PAs), on the other hand, show significant depletions under N− in *E. oleoabundans,* while the previous studies in other species have not reported any changes in their respective levels [27, 28, 49]. In addition, under N−, *E. oleoabundans* resulted in depletion of phosphatidylglycerols (PGs) and FAs, while in *Chlorella* sp. PGs [27] and in *Nannochloropsis* sp. FAs were accumulated [49]. We note that the data on changes in lipid composition under P− are limited compared to N−, and while we did not observe significant changes in total lipid levels in *E. oleoabundans* under P−, in *Chlorella* sp., there was an increase in lipid content [43].

### Biomass calculations, extraction, and normalization of metabolites and extraction efficiencies

Prior to LC–MS analysis, dried biomass values of all samples were calculated for 10 mL aliquots of algal culture, and extracted microalga were normalized to 9.20 mg of biomass/mL in chloroform (Additional file 1: Table S1, the normalized value was calculated based on the measured biomass provided in “Determination of optical density, nitrate and phosphate concentrations in the media, dry biomass, and lipid composition” section). The results of the measurement showed a range of biomass from 0.214 mg/mL (in one of the biological replicates for N − 2 condition) up to 1.478 mg/mL (in one of the biological replicates for P + 7 condition). These measured biomasses and calculated normalization values are shown in Additional file 1: Table S1.

To ensure that lipids were extracted similarly within all experimental conditions and reliable downstream analyses were conducted, we investigated the extraction efficiencies of two internal standards spiked into the samples during different stages of metabolite extraction at the most extreme conditions, i.e., nutrient-deplete on day 2 and -replete on day 10 (Additional file 1: Figure S1A, B). We found that the extraction efficiencies of TAG (19:0/19:0/19:0) were close to 100% and showed no significant difference (Additional file 1: Figure S1B). On the other hand, we found modest differences in the extraction efficiency of oleic acid-*d*9 (62–99%, Additional file 1: Figure S1A), which we attributed to the fact that oleic acid-*d*9 was spiked at a very early point during extraction and, thus, was slightly affected by sample handling. Overall, the high recovery values for these standards in different experimental conditions showed that the extraction protocol was effective and reproducible for the metabolomics analyses to be performed.

### Untargeted metabolomics of the hydrophobic metabolites

**Fig. 2:**
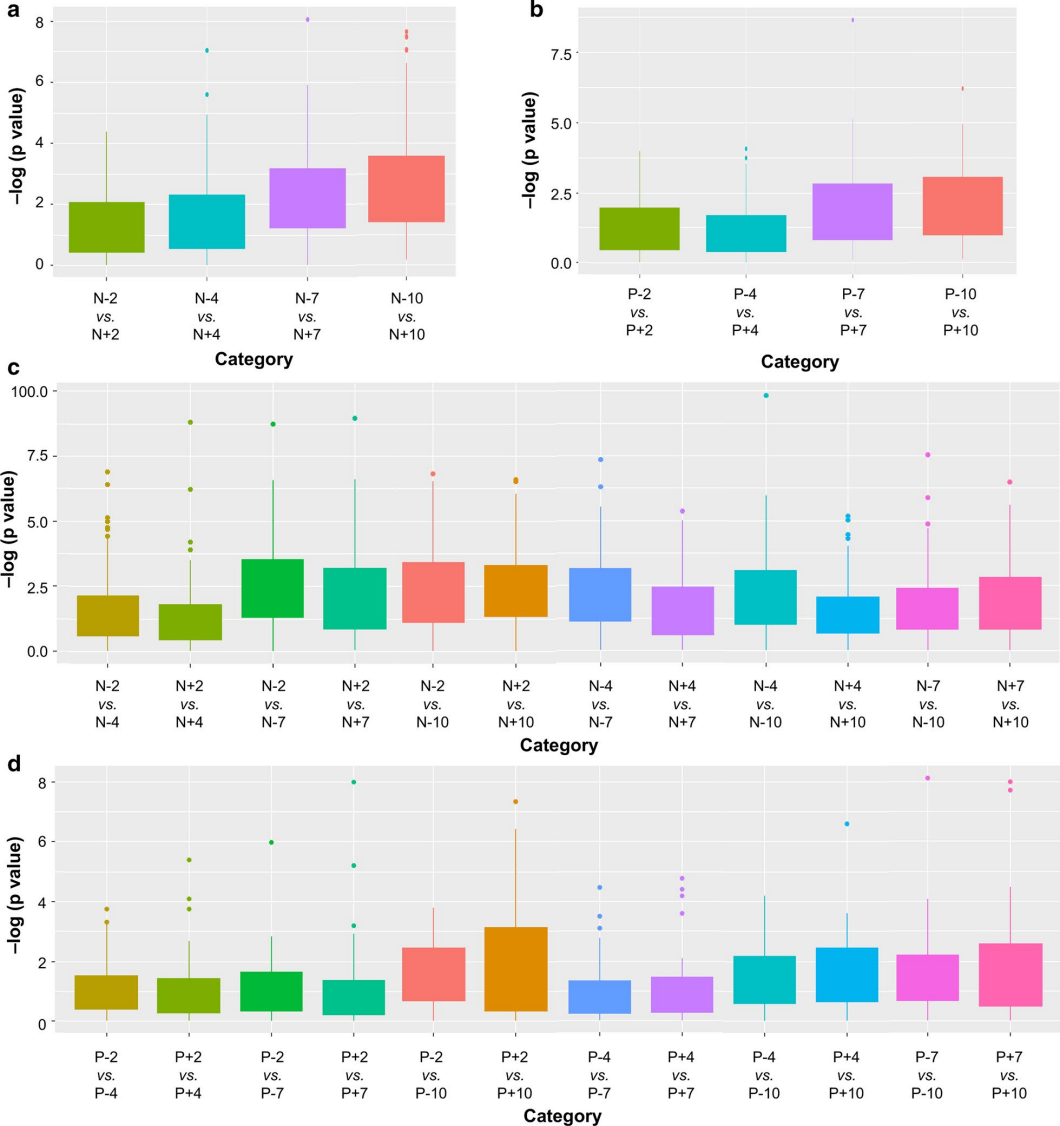
Nutrient and temporal analysis of significant *m*/*z*’s detected in *E. oleoabundans*. **a** − log(*p* value) of N− vs. N+ of the same time period. **b** − log(*p* value) of P− vs. P+ of the same time period. **c** − log(*p* value) of varying time points of the same nutrient conditions (N− or N+). **d** − log(*p* value) of varying time points of the same nutrient conditions (P− or P+)

The samples grown under varying nitrate (N− vs. N+) and phosphate (P− vs. P+) conditions were analyzed separately. Two independent profiling experiments with three biological replicates (*n* = 3, highlighted in gray in Additional file 1: Table S1) from all 16 sampling conditions were performed to identify reproducible changes in the hydrophobic metabolite composition (sampling conditions are listed in Additional file 1: Figure S1C). The data analysis for the initial untargeted profiling was performed following a procedure we describe in detail elsewhere ([35], Additional file 1: Figure S2). We focused on *m*/*z*’s that were present in 100% of the biological replicates in at least one experimental condition and showed reproducible significant changes (*p* < 0.01, see “Experimental methods”, “Untargeted analysis and LC–MS/MS identification” section for details, Additional file 1: Figure S2). These analyses resulted in 86 and 39 *m*/*z*’s that showed a significant differentiation under varying nitrate and phosphate conditions over time, respectively (Fig. 2, Additional file 1: Figure S2 and Table S3). The greater number of *m*/*z*’s identified under N− vs. P− was consistent with the more profound differences in overall lipid contents under these conditions (Fig. 1d).

We were also interested in pairwise comparisons between experimental conditions and calculated *p* values for both nutrient variations and different growth phases (Fig. 2a–d). Figure 2a, b shows the *p* values for the comparison of the same growth phase (i.e. days 2, 4, 7 and 10) under N− vs. N+ (Fig. 2a) and P− vs. P+ (Fig. 2b), respectively. When different nutrient conditions at the same growth period were compared to each other, there was increased significance as the time period increased from day 2 to day 10 for both nitrate and phosphate variations (Fig. 2a, b, respectively), which suggested at the biochemical level that differences in metabolite composition increased from the early exponential to late stationary growth period in *E. oleoabundans*. This observation was in line with the differences in lipid composition in Fig. 1d which showed that the lipid composition had the greatest difference, especially for nitrate variation at day 10. Comparison of the two different growth periods under the same nutrient condition showed that the differences between day 2 vs. day 10 and day 2 vs. day 7 were the most significant under nitrate variation, while the difference between day 2 vs. day 10 was the most significant for phosphate variation (Fig. 2c, d, respectively).

**Fig. 3:**
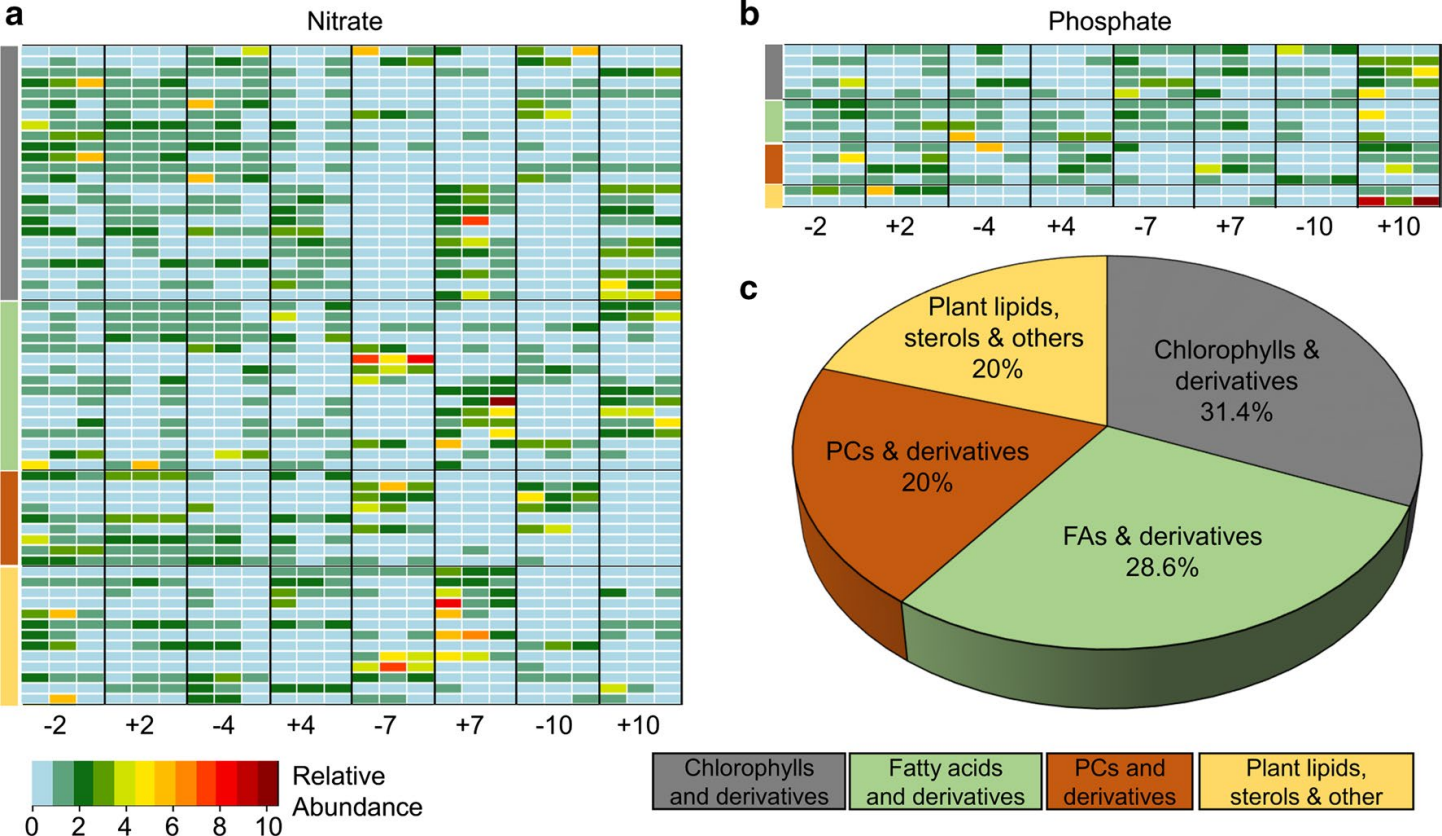
Hydrophobic metabolites identified under untargeted profiling in *E. oleoabundans.* (N− vs. N+ and P− vs. P+ during different growth periods: day 2, early exponential phase; day 4, late exponential phase; day 7, early stationary phase; and day 10, late stationary phase). **a** Relative abundances of annotated *m*/*z*’s that showed significant variations between varying nitrate conditions and time periods. **b** Relative abundances of annotated *m*/*z*’s that showed significant variations between varying phosphate conditions and time periods. **c** Distribution of annotated *m*/*z*’s presented in **a**, **b** based on metabolite family

For the untargeted hydrophobic metabolite sample set, we carried out MS/MS experiments to identify the *m*/*z*’s showing significant differentiation and annotated 70 successfully (55 out of 86 under nitrogen variation and 15 out of 36 under phosphorus variation, Fig. 3a, b and Additional file 1: Table S3). The relative abundances of the annotated *m*/*z*’s are presented in Fig. 3a, b for nitrate and phosphate conditions, respectively. A majority of these *m*/*z*’s (~ 31%) were identified as chlorophylls and their derivatives (shown in gray in Fig. 3a, b, and c) which showed depletion in N − 7, N − 10, and P − 10 conditions. In this analysis, ~ 29% of the identified *m*/*z*’s were simple lipids such as FAs and their common hydroxylated derivatives [50] shown in green in Fig. 3a, b, and c, 20% were PCs and their derivatives (shown in brown in Fig. 3a, b, and c), 20% were comprised of other lipid families such as plant lipids (MGMGs and DGDGs), phytosterols, a monoacylglycerol (MAG), a phytosphingolipid, a quinone derivative, and short peptides (shown in yellow in Fig. 3a, b, and c, Additional file 1: Table S3).

### Targeted analysis of endogenous lipids

**Fig. 4:**
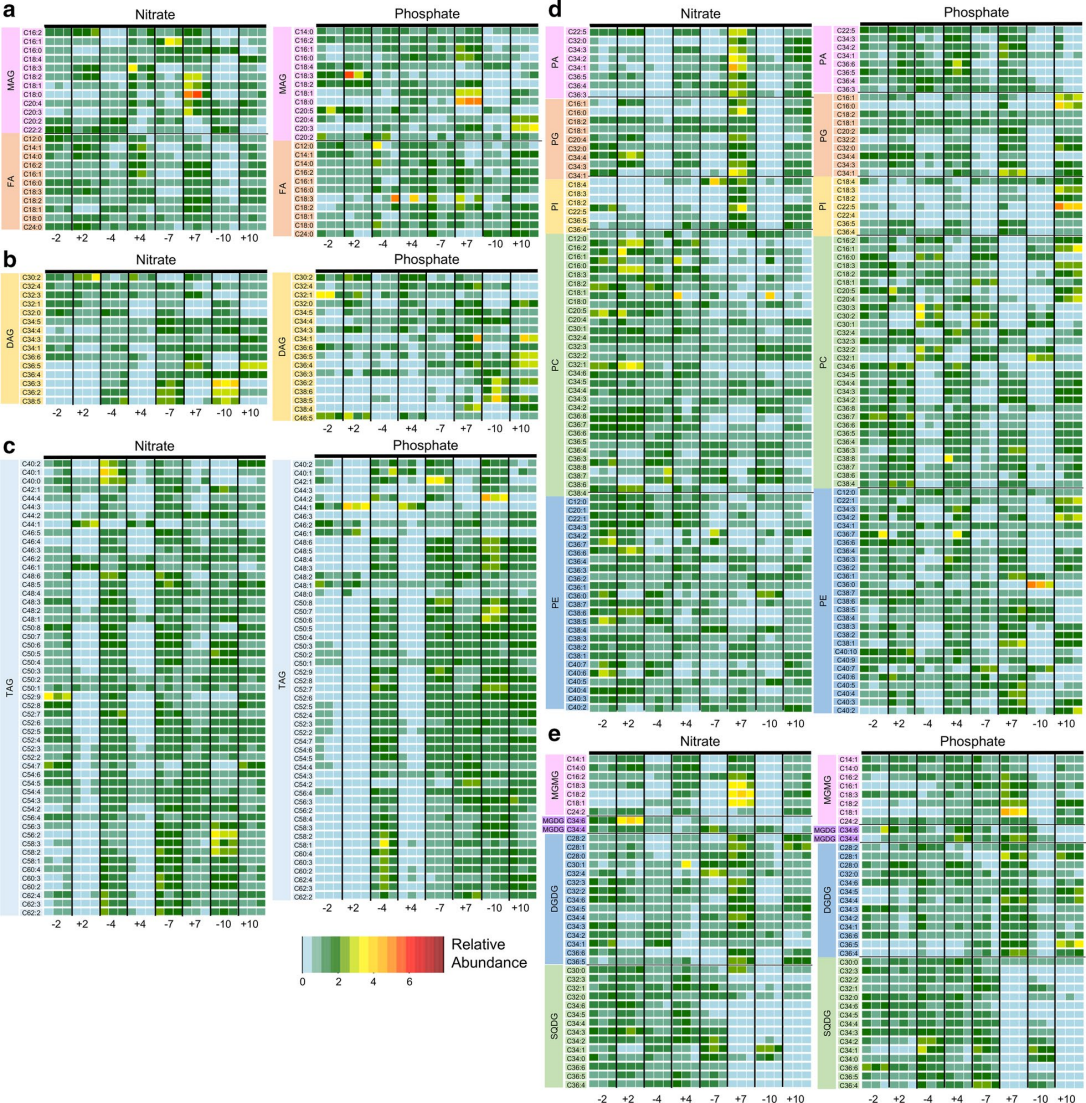
Targeted analysis results of lipids detected and identified in *E. oleoabundans.* Relative abundances of **a** monoacylglycerols (MAGs) and fatty acids (FAs), **b** diacylglycerols (DAGs), **c** triacylglycerols (TAGs), **d** phospholipids **e** monogalactosylmonoacylglycerols (MGMGs), monogalactosyldiacylglycerols (MGDGs), digalactosyldiacylglycerols (DGDGs), and sulfoquinovosyldiacylglycerols (SQDGs)

Untargeted analyses allowed us to obtain information on changes in hydrophobic metabolite composition at a global level in *E. oleoabundans* under nitrate and phosphate variations over different growth phases. To better understand the biochemical regulations that are coupled to overall lipid accumulation (Fig. 1d) and identify the lipid groups that contribute to this accumulation at a molecular level, we conducted targeted analyses on representative members from major lipid families. In particular, we focused on FAs, MGMGs, MGDGs, DGDGs, SQDGs, PAs, PGs, and phosphatidylinositols (PIs) in ESI− mode and MAGs, diacylglycerols (DAGs), TAGs, phosphatidylcholines (PCs), and phosphatidylethanolamines (PEs) in ESI+ mode (Additional file 1: Table S3). We targeted lipids from these families with carbon chains ranging from 12 to 24 carbons in length and different unsaturation states. In Fig. 4, we only report lipids that were detected. After confirming their identity by LC–MS/MS, we investigated the changes in abundances under all experimental conditions. Their raw abundances, *m*/*z*’s, adducts, and observed retention times are provided in Additional file 1: Table S3.

#### Changes in fatty acids and glycerolipids

One of the lipid families that showed alterations at the global level, especially under nitrogen limitation, was FAs and their hydroxylated derivatives. To obtain detailed information regarding the changes in FAs and how these changes might impact other downstream lipids at the molecular level, we targeted different FAs and glycerolipids. We found significant changes in their abundances not only over time but also across nutrient sources (nitrate and phosphate). For instance, specific FAs (i.e., C14:1, C16:2, C16:1, and C18:3) were depleted in N− over time, whereas they remained similar in P− and showed some accumulation in P+ (Fig. 4a). Overall, FA accumulation was highest on N + 7 and P + 10. Deactivation of de novo fatty acid biosynthesis-related enzymes including acyl-CoA carboxylase (ACC) [23, 30, 51, 52], acyl-CoA synthetase (ACS) [52], ATP citrate lyase (ACL), fatty acid synthetase (FAS), and acetyl-CoA C-acetyltransferase (ACAT) [52] under nutrient deprivation over time may be involved in the changes which we observed in FAs. Alternatively, activation of downstream phospholipid or glycerolipid biosynthesis can also cause these depletions.

An intermediate lipid generated during glycerolipid biosynthesis, MAGs, showed no significant changes in N− or N+ but showed a dynamic regulation under P−. Their levels first increased at day 4 and day 7, followed by a depletion at day 10, which suggests that at the later time points, these lipids were either diverted to different lipids or degraded to replenish free fatty acid pools (Fig. 4a). With respect to DAGs, a significant proportion detected in samples (i.e. C32:1, C32:0, C36:3, C36:2, and C38:5) was accumulated in N − 7 and N − 10. This was also the case for P−, where DAGs mostly accumulated over time (Fig. 4b). Finally, TAGs, which are synthesized via esterification of DAGs, showed accumulation over time at all analyzed conditions. An interesting observation was *E. oleoabundans* accumulated longer TAGs under N− and P− (Fig. 4c), which are usually characteristics of marine microalgae species [53]. Overall, these results support the overall tendency to divert simpler lipids (i.e., FAs, MAGs, and DAGs) for TAG biosynthesis in *E. oleoabundans* similar to most other oleaginous microalgae species.

#### Changes in phosphatidylglycerols and other glycerophospholipids

Phospholipids are important precursors for different modifications including glycosylation. In this study, we targeted different phospholipids, glycerophospholipids, and their lysolipids (i.e., single acylation at the glycerol backbone), and found that there were not only significant changes between different nutrient conditions (N− vs. N+ and P− vs. P+) but also between growth phases.

PAs, the simplest glycerophospholipids, detected in *E. oleoabundans* extracts were depleted in N− and in P − 10 and P + 10 (Fig. 4d). PGs and PIs were also depleted in N− and P− (Fig. 4d). The depletion of PGs in N− was surprising, because the previous studies in other green microalgae reported their depletion under N+ [27, 28]. PCs, another sub-class of glycerophospholipids, were depleted in N− and P− over time (i.e., C34:2–34:6, C36:3–36:8, and C38:4–38:8), whereas lysoPCs did not follow a specific trend (Fig. 4d). This was also the case for PEs: some PEs (i.e., C34:2–34:3, C36:1–36:7, C38:1–38:3, C40:9–40:10, and C40:2–40:4) were depleted, while others (i.e., C36:0, C38:4–38:7 and C40:5–40:7) were accumulated in P− (Fig. 4d).

Based on the changes which we observed in phospholipids and glycerophospholipids, we propose that when the nutrient levels are depleted in *E. oleoabundans*, there is also a depletion of PAs and PGs in upstream phospholipid biosynthesis. It is also possible that FAs and DAGs were used to “replenish” these glycerophospholipid pools. Since PAs can also functions as signaling molecules [54], its depletion might have negatively affected the synthesis of other lipid families and/or cellular homeostasis. These observations need to be further verified with proteomic and transcriptomic characterizations.

#### Changes in chlorophylls and PGs under nitrate limitation

Under nutrient-limited conditions, cellular proliferation rate and overall photosynthetic activity in microalgae decrease [20, 55]. We observed a gradual depletion of chlorophylls from N − 2 to N − 10 and their gradual accumulation from N + 2 to N + 10, which constituted the majority of the changes that we identified based on untargeted analysis (Fig. 3). This could be due to the fact that under N−, microalgal cells were deprived of nitrogen, one of the building blocks of the porphyrin core. We observed a similar trend in PGs (Fig. 4d), which are important structural lipids found in the thylakoid membrane where photosynthetic reactions and electron transport take place [56–59]. For ease of comparison, we summarize these changes in Additional file 1: Figure S3A, B. Based on these observations, it is highly likely that, in addition to the decreased levels of chlorophyll and derivatives under nutrient limitation over time, the depletion of PGs compromises the integrity of thylakoid membranes [20] and, thus, the photosynthetic efficiency in the chloroplasts decreases under these conditions.

#### Changes in sulfoquinovosyldiacylglycerols and other plant lipids

The plant lipidome is rich and complex, encompassing many unique plant-specific lipids [56, 58]. To characterize this diversity, we targeted different plant lipids and studied the changes in their levels under N−, N+, P−, and P+ during different growth stages. Particularly, we targeted MGMGs, MGDGs, DGDGs, and SQDGs and found that most of these lipids were differentially regulated between different growth periods and between different nutrient conditions (N− vs. N+ and P− vs. P+) in *E. oleoabundans*.

MGMGs are the simplest plant lipids with one sugar moiety linked to a MAG. These lipids are depleted starting from late exponential (day 4) to late stationary growth (day 10) phase under N− (Fig. 4e). Even though depletion under P− was observed at late stationary phase, this depletion was preceded by a slight accumulation at early stationary phase (day 7). MGDGs, DAGs with one sugar group at the glycerol backbone, on the other hand, did not show any significant changes between N− vs. N+, P− vs. P+, or different growth phases (Fig. 4e). DGDGs, which are MGDGs with an additional sugar group, show a similar trend as MGMGs with a depletion starting from late exponential (day 4) to late stationary (day 10) phase under N− and depletion from early (day 7) to late stationary (day 10) phase in P− (Fig. 4e).

Next, we analyzed the levels of SQDGs, which are derivatives of MGDGs with sulfonated sugar moieties [56, 60]. These lipids are found in photosynthetic plants, algae, and other organisms, and are located in the thylakoid membranes [56]. The previous studies have shown their accumulation under N+ [26–28], as well as their degradation under sulfur depletion to repurpose sulfur for the synthesis of new proteins in *C. reinhardtii* [61, 62]. Our study showed that SQDGs remain at sustained levels until early stationary phase. After this time point, they show an overall depletion both under nutrient replete and deplete conditions; however, surprisingly, their depletion is more pronounced under N+ and P+ compared to N− and P− at late stationary phase (day 10) (Fig. 4e). We inferred from these results that based on the decreased nutrient concentration at day 10 for N+ and P+ ([NO_3_^−^] = 153.7 µM, N+; [PO_4_^3−^] = 247.7 µm, P+), *E. oleoabundans* might be utilizing SQDGs to provide the necessary building blocks (such as sulfur) for protein synthesis and other components that are necessary to maintain cellular homeostasis.

### Changes in saturated fatty acid (SFA), monounsaturated fatty acid (MUFA), and polyunsaturated fatty acid (PUFA) fatty acids (FAs) as compared to phosphatidylglycerols (PGs), digalactosyldiacylglycerols (DGDGs), and sulfoquinovosyldiacylglycerols (SQDGs)

When comparing the levels of different lipids, FAs are of great interest, since they are the building blocks for the synthesis of downstream lipids. To obtain insight on the changes of FAs and other lipids based on their unsaturation state, we produced a heatmap showing relative abundances of different FAs, PGs, DGDGs, and SQDGs (Additional file 1: Figure S4). We grouped different lipids as saturated fatty acid—(SFA), monounsaturated fatty acid—(MUFA), and polyunsaturated fatty acid—(PUFA) containing lipids. To properly classify lipids as monounsaturated or polyunsaturated lipids, we grouped those with a total of one double bond on the acyl chains as MUFAs and those with a total of three or more double bonds as PUFAs.

MUFAs and PUFAs when grown under N− decrease in abundance over time as can be seen by the decrease in their relative abundance during day 7 and day 10 (early and late exponential phase), while SFAs gradually decrease in abundance over time both under N+ or N−. Comparing the changes in FAs under N− vs. N+ shows that C14:1, C16:1, and C18:1 FAs are accumulated in N + 4 and N + 7 and N + 10 (late exponential; early and late stationary phases) as compared to the N − 4, N – 7, and N − 10 growth conditions. While these trends are apparent under nitrate growth conditions, there seems to be no significant alterations in FAs over time in phosphate growth conditions.

SFA-, MUFA-, and PUFA-PGs decrease in abundance over time when grown under either N− or P−, while SFA- and MUFA-PGs increase in abundance under N+ and P+ at early and late stationary phases.

The plant lipids which we analyzed show decreased levels under N−. Specifically, SFA- and PUFA-DGDGs when grown under N+ increase in abundance over time, while MUFA- and PUFA-DGDGs when grown under P+ increase in abundance over time. However, PUFA-DGDGs when grown under N− decrease in abundance over time. SQDG, on the other hand, exhibits differing trends: MUFA-SQDGs increase over time under N− and P−, and were depleted under P+. PUFA-SQDGs when grown under all four growth conditions decrease in abundance over time. Overall these trends in PGs, DGDGs, and SQDGs could be attributed to the gradual depletion over time of the SFAs when grown under N+ and N− and the depletion over time of the MUFAs and PUFAs when grown under N−. However, based on our data, we are limited in explaining how *E. oleoabundans* utilized FAs for the production of downstream lipids under nutrient limitation. Though the biochemical machinery that controls the incorporation of different FAs to other complex lipids is well defined in plants, this seems to be more complex in green microalgae [48].

### Overall analysis of hydrophobic metabolites

Lipidomics studies carry great importance and have been performed on several microalgae species grown under nutrient replete vs. deplete conditions. A majority of these analyses have only focused on TAG accumulation and how nitrogen limitation affects its synthesis [63]. Under nitrogen deprivation, MGDGs, DGDGs, SQDGs, and PGs were depleted, while TAGs were accumulated in *C. reinhardtii* [28]. Similarly, in *Chlorella* sp., MGDGs, DGDGs, SQDGs, PEs, and PCs were depleted, while PGs and TAGs were accumulated under nitrogen deprivation [27]. Another model green microalga, *Nannochloropsis* sp., when grown under nitrogen deprivation, showed depletions of MGDGs, DGDGs, SQDGs, PCs, PGs, and PIs, the accumulation of TAGs, and no significant trends for PEs [26].

In this study, we carried out a time-series characterization of the effect of different nutrient limitation conditions on lipid composition in a highly adaptable and oleaginous *E. oleoabundans* non-model microalgae. Using a combination of targeted and untargeted lipidomics, we analyzed the levels and detected 377 unique lipids which allowed us to obtain information at the global and metabolic pathway level. In particular, we analyzed the effects of N− and P− during early exponential, late exponential, early stationary, and late stationary phases on lipid composition. This is the first comprehensive time-series analysis of N− vs. N+ and P− vs. P+ in a non-model green microalga which provided a dynamic picture of the changes in lipid families with respect to cellular growth.

**Fig. 5:**
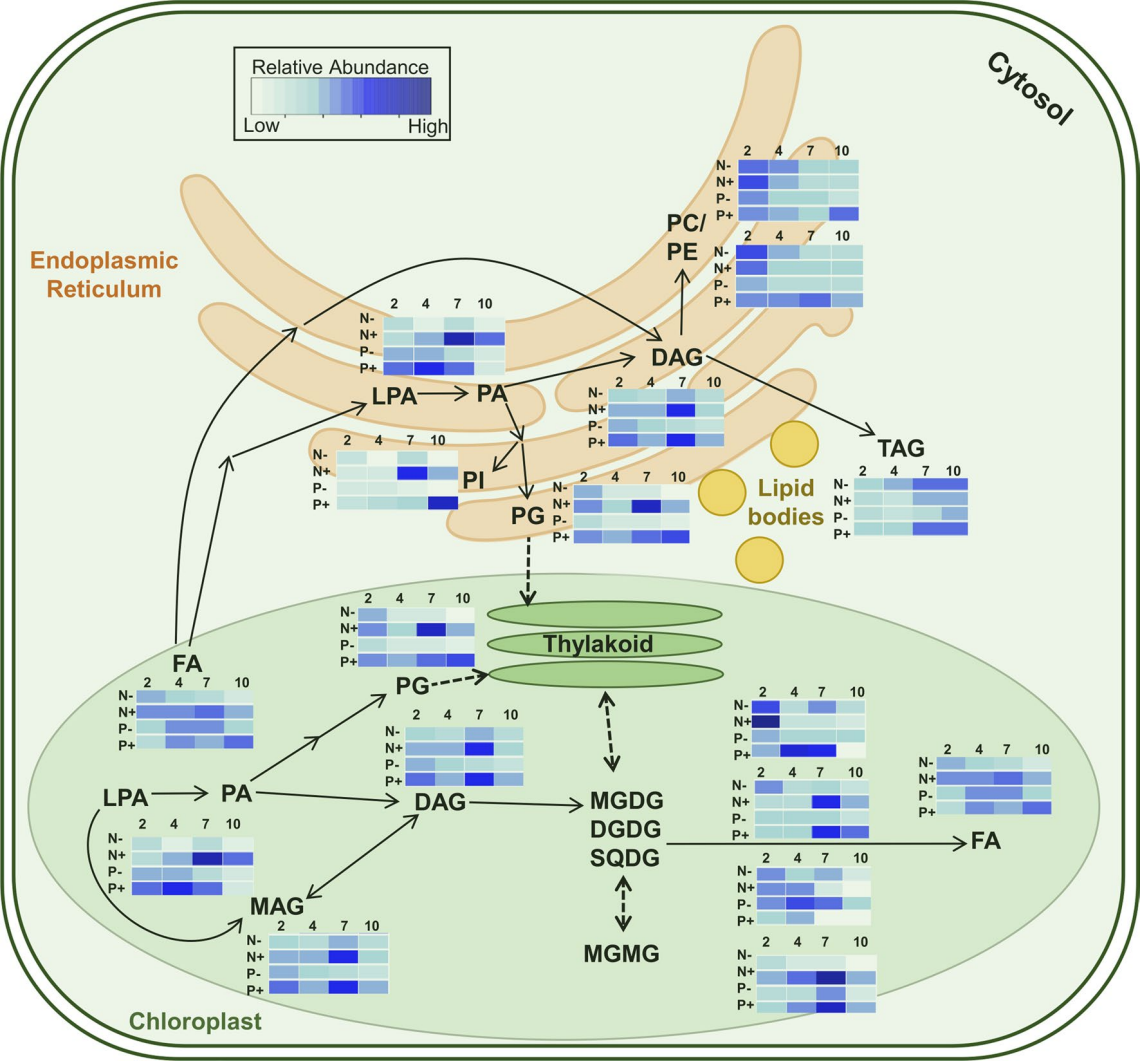
Data-based schematic of plant lipid production pathways. Shown are the averaged relative abundances of different lipid families in different growth conditions (N−, N+, P−, and P+) and at different growth periods (day 2, early exponential phase; day 4, late exponential phase; day 7, early stationary phase; and day 10, late stationary phase)

A summary of global changes in lipid composition is provided in Fig. 5. To illustrate the time-dependent changes in a given lipid family under different conditions, each box represents the average relative abundances of all lipids that we targeted in a given family. Based on this, the optimal growth conditions for each lipid family are as follows: N − 2 for PEs, N + 2 for PCs and MGDGs, P − 4 for SQDGs, N + 7 for PAs, PGs, and FAs, P + 10 for PIs, either N + 7 or P + 7 for DAGs, DGDGs, and MGDGs, and N − 7 or N − 10 or P + 7 or P + 10 for TAGs. We show that a strong accumulation of TAGs, critical precursors for biodiesel production, and depletion of chlorophylls occur under deprivation as cells reach late stationary phase (Fig. 5).

Multiple enzymes might contribute to the changes in glycerolipids. These include glycerol-3-phosphate O-acyltransferase (GPAT) [52, 64], 1-acyl-sn-glycerol-3-phosphate O-acyltransferase (AGPAT) [51, 52], lysophosphatidic acid acyltransferase (LPAAT) [64], phosphatidic acid phosphatase (PP or PAP) [51, 52, 64], and diacylglycerol acyltransferase (DGAT-1 and DGAT-2) [23, 30, 51, 52, 64] and phospholipid: diacylglycerol acyltransferase (PDAT) [23, 52], which have been correlated to changes in the MAG, DAG, and TAG abundance trends that are seen when grown under N− and P− as compared to N+ and P+. We envision that the activation of DGAT-1 and DGAT-2 plays important roles in the strong accumulation of TAG accumulation may stem from an over expression of DGAT-1 or 2.

In addition, phospholipids showed regulation based on acyl chain composition, except for PGs which gradually decreased over time in nutrient-depleted conditions (Fig. 5). This depletion of PGs most likely affected the chloroplast and thylakoid membrane structure and the production of chlorophylls, which in turn decreased overall photosynthetic efficiency of *E. oleoabundans* as supported by the growth profiles (Fig. 1a). Finally, we observed a non-conventional regulation of SQDGs, which were depleted under N+ and P+ during the stationary phase (Fig. 5). We reason that this could be due to a switch in metabolism to utilize sulfur for the synthesis of essential biomolecules for cellular survival under nutrient stress. Intriguing questions remain to be answered on the biochemical machinery that regulates these changes. For instance, UDP-sulfoquinovose synthase (SQD1) [23], sulfoquinovosyldiacylglycerol synthases, (SQD2 or SQD3) [23], monogalactosyldiacylglycerol synthases (MGD1 or MGDGS) [23, 64], and digalactosyldiacylglycerol synthases (DGD1 or DGDGS) [23, 64] could be involved in the changes in plant lipids which we observed under N− or P−. Understanding the role of individual enzymes and biosynthetic pathways will be the key to elucidate important metabolic transitions under nutrient deprivation.

## Conclusions

In this work, we conducted the first time-series characterization of the changes in the lipidome under nutrient deprivation in a non-model oleaginous microalga. Integrating targeted and untargeted analyses, we captured the temporal regulation and overall accumulation of TAGs from early exponential to late stationary phases, which is advantageous for biodiesel production in *E. oleoabundans*, as well as novel regulations including ones in structural and plant-specific lipids. These results clearly show that there is a complex regulation of lipids under nutrient variation with respect to cellular growth in the microalga species. Future studies that include information on more species and experimental conditions elucidating this complexity will provide invaluable insights into the optimized production of value-added products including biodiesel in green microalgae.

## Additional file

**Additional file 1.** Three additional figures and two additional tables.

## Abbreviations

TAGs: triacylglycerols
DGDGs: digalactosyldiacylglycerols
SQDGs: sulfoquinovosyldiacylglycerols
PGs: phosphatidylglycerols
FAs: fatty acids
HR-MS: high-resolution mass spectrometry
LC–QToF-MS: liquid chromatography–quadrupole time-of-flight mass spectrometry
DCW: dry cell weight
N−: nitrogen deplete
P−: phosphorus deplete
N+: nitrogen replete
P+: phosphorus replete
LC–MS: liquid chromatography–mass spectrometry
HPLC: high-performance liquid chromatography
ESI: electrospray ionization
ESI+: positive mode
ESI−: negative mode
NO_3_^−^: nitrate
PO_4_^3−^: phosphate
PBRs: photobioreactors
MPP: Mass Profiler Professional
ANOVA: analysis of variance
FDR: false discovery rate
LC–MS/MS: liquid chromatography–tandem mass spectrometry
MS/MS: tandem mass spectrometry
MGMGs: monogalactosylmonoacylglycerols
MGDGs: monogalactosyldiacylglycerols
PAs: phosphatidic acids
PIs: phosphatidylinositols
MAGs: monoacylglycerols
DAGs: diacylglycerols
PCs: phosphatidylcholines
PEs: phosphatidylethanolamines
SFAs: saturated fatty acids
MUFAs: monounsaturated fatty acids
PUFAs: polyunsaturated fatty acids
ACC: acyl-CoA carboxylase
ACS: acyl-CoA synthetase
ACL: ATP citrate lyase
FAS: fatty acid synthetase
ACAT: acetyl-CoA C-acetyltransferase
GPAT: glycerol-3-phosphate O-acyltransferase
AGPAT: 1-acyl-sn-glycerol-3-phosphate O-acyltransferase
LPAAT: lysophosphatidic acid acyltransferase
PP: phosphatidate phosphatase
DGAT-1 and DGAT-2: diacylglycerol acyltransferase
PDAT: phospholipid: diacylglycerol acyltransferase
SQD1: UDP-sulfoquinovose synthase
SQD2 or 3: sulfoquinovosyldiacylglycerol synthase
MGD1 or MGDGS: monogalactosyldiacylglycerol synthase
DGD1 or DGDGS: digalactosyldiacylglycerol synthase
