# Supplementary material for: Time-series lipidomic analysis of the oleaginous green microalga species Ettlia oleoabundans under nutrient stress
Source: Biotechnol Biofuels. 2018 Feb 6;11:29. doi: 10.1186/s13068-018-1026-y (PMC5800086; doi:10.1186/s13068-018-1026-y)
Supplement: Supplementary file 1 — Additional file 1. Three additional figures and two additional tables. [file 13068_2018_1026_MOESM1_ESM.zip › Matich2017_SI_0130.pdf]

Additional file 1

Time series lipidomics analysis of the oleaginous green microalga species *Ettlia oleoabundans* under nutrient stress

E. K. Matich[1], M. Ghafari[2], E. Camgoz[3], E. Caliskan[4], B. A. Pfeifer[5], B. Z. Haznedaroglu[4]*, and G. E. Atilla-Gokcumen[1]*

[1] Department of Chemistry, University at Buffalo, The State University of New York (SUNY), Buffalo, NY USA 14260

[2] Department of Civil, Structural and Environmental Engineering, University at Buffalo, The State University of New York (SUNY), Buffalo, NY USA 14260

[3] Department of Chemical and Biological Engineering, Koc University, Istanbul, Turkey 34450

[4] Institute of Environmental Sciences, Bogazici University, Istanbul, Turkey 34342

[5] Department of Chemical and Biological Engineering, University at Buffalo, The State University of New York (SUNY), Buffalo, NY USA 14260

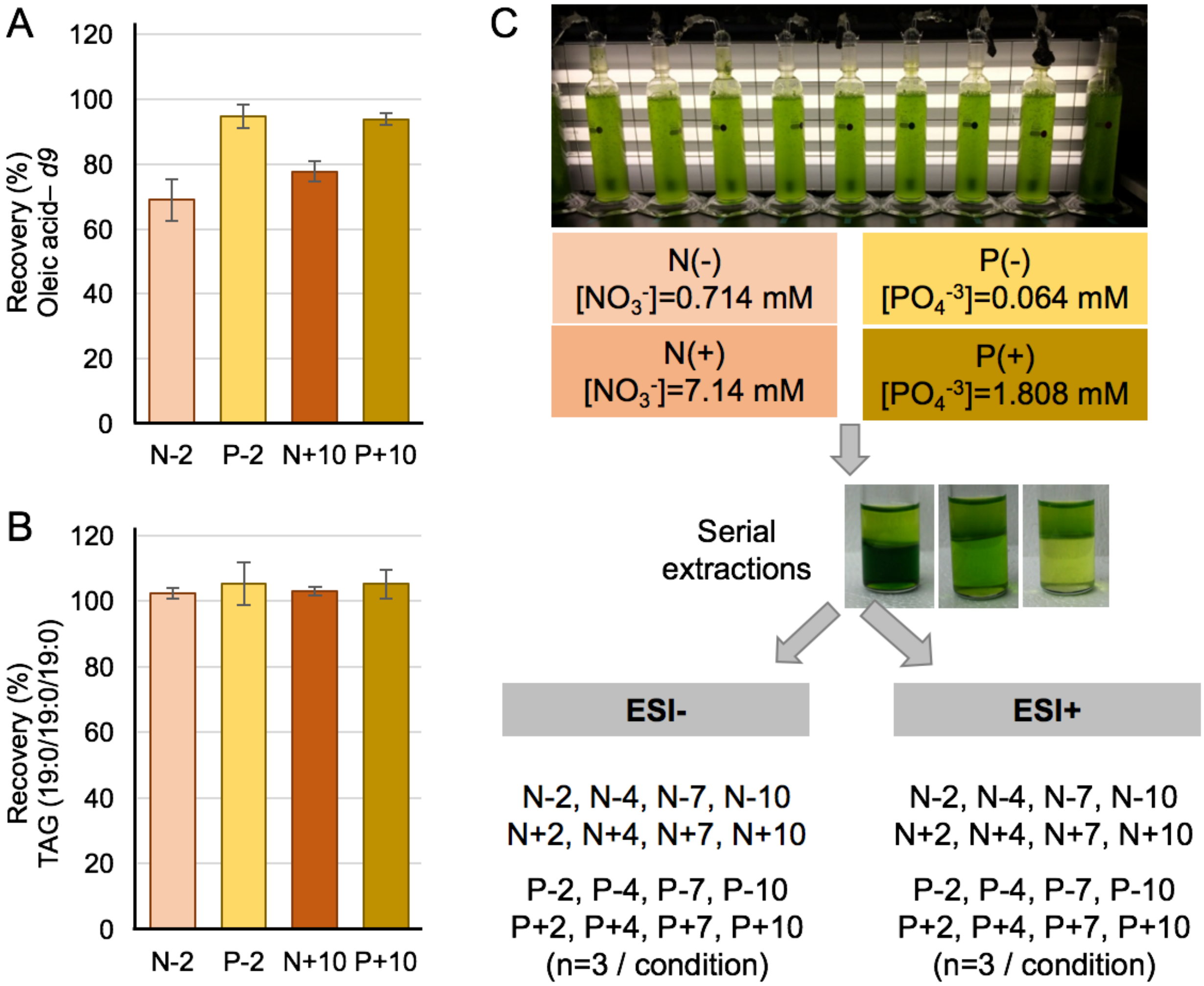

Figure S1. Extraction efficiency, growth, extractions and independent analysis. (A) Representative graph of extraction efficiency ([measured concentration/theoretical concentration]*100) of oleic acid – *d*9 in *E. oleoabundans* samples grown in different nitrate and phosphate concentrations for different periods of time (conditions and time periods which had the biggest difference in biomass are shown) calculated based on a calibration curve. (B) Representative graph of extraction efficiency ([measured concentration/theoretical concentration]*100) of TAG (19:0/19:0/19:0) in *E. oleoabundans* samples grown in different nitrate and phosphate concentrations for different periods of time (conditions and time periods which had the largest difference in biomass are shown) calculated based on a calibration curve. (C) Schematic for microalgae growth (including starting nitrate and phosphate concentrations in growth media); sample extractions and LC-Q-ToF data acquisition performed in ESI- and ESI+ modes.

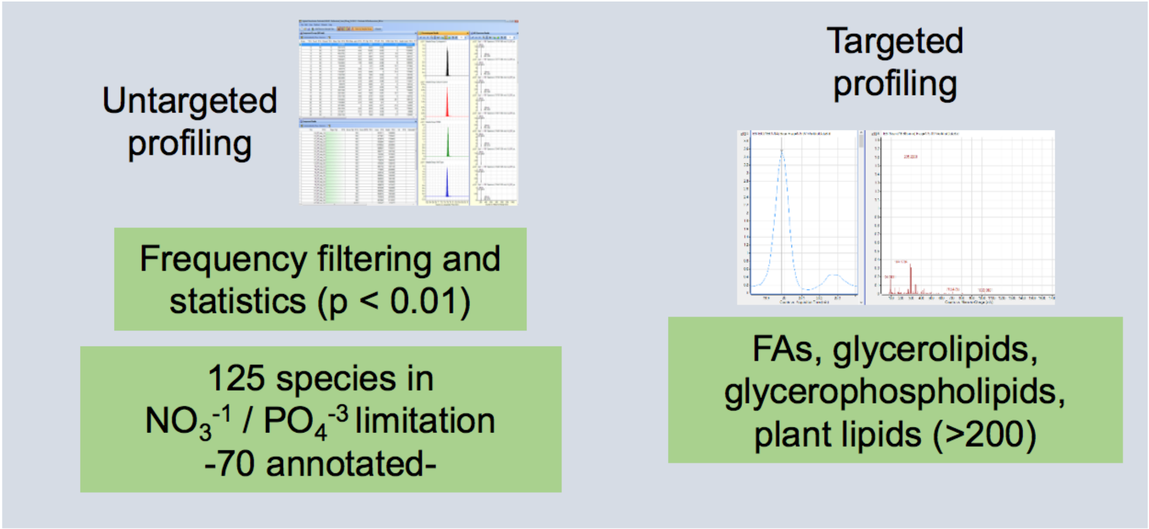

Figure S2. LC-QTOF analysis workflow

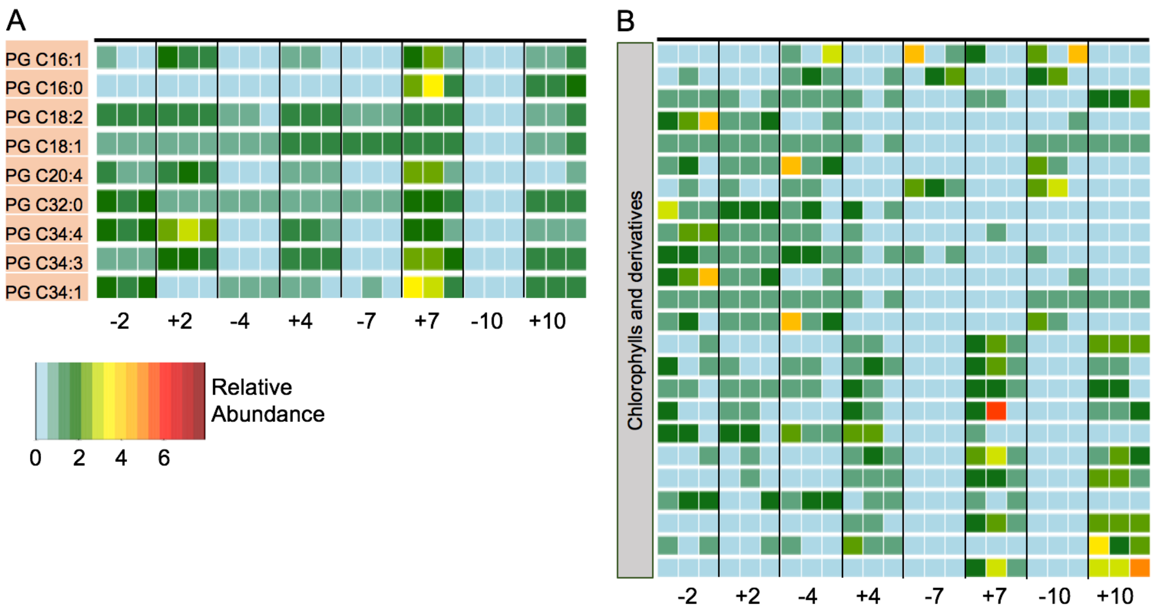

Figure S3. Relative abundance of PGs and chlorophylls in N- and N+

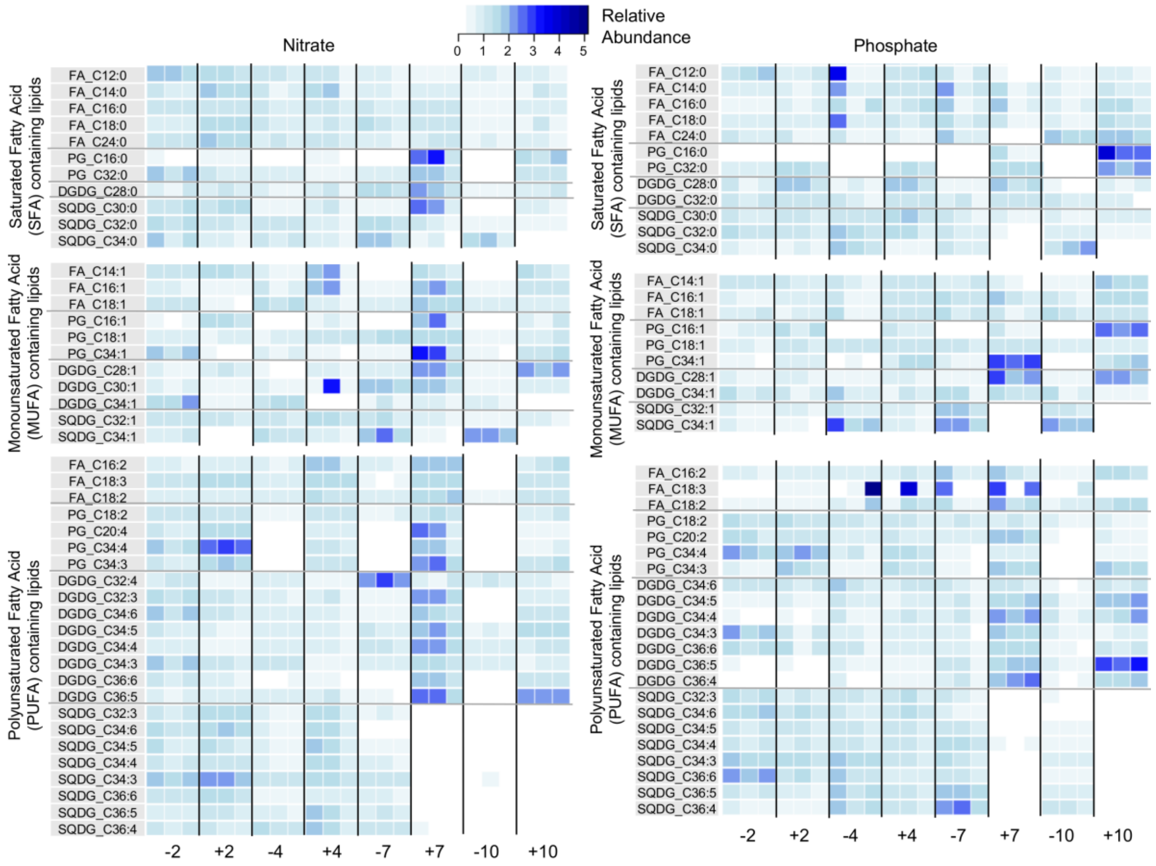

Figure S4. Changes in saturated fatty acid (SFA), monounsaturated fatty acid (MUFA) and polyunsaturated fatty acid (PUFA) fatty acids (FAs) as compared to phosphatidylglycerols (PGs), digalactosyldiacylglycerols (DGDGs) and sulfoquinovosyldiacylglycerols (SQDGs).

Relative abundance heat maps of FAs, PGs, DGDGs, and SQDGs separated into those with SFAs, MUFAs and PUFAs.

Table S1. Dry biomasses per 10 mL of algal growth along with calculated normalization values.
Biological replicates used in lipidomics analyses are highlighted in gray.

Cond.	Reactor	biomass (mg/ 10 mL)	mL	mg per pellet	mg per 1/8 of pellet	mg/mL after norm	mL re-suspend
N-2	A	4.06	340	138.04	17.26	9.20	1.86
	B	3.80	335	127.30	15.91	9.20	1.72
	C	**2.14**	350	74.90	9.36	9.20	1.00
	D	4.21	335	141.04	17.63	9.20	1.91
	E	4.64	331	153.58	19.20	9.20	2.09
N+2	F	4.93	344	169.59	21.20	9.20	2.28
	G	5.45	340	185.30	23.16	9.20	2.50
	H	5.28	326.5	172.39	21.55	9.20	2.35
	I	4.15	333	138.20	17.27	9.20	1.87
	J	3.21	347	111.39	13.92	9.20	1.49
N-4	A	5.27	333	175.49	21.94	9.20	2.38
	B	4.86	360	174.96	21.87	9.20	2.32
	C	5.05	349	176.25	22.03	9.20	2.36
	D	5.63	333	187.48	23.43	9.20	2.54
	E	5.23	334	174.68	21.84	9.20	2.37
N+4	F	6.33	340	215.22	26.90	9.20	2.90
	G	7.33	343	251.42	31.43	9.20	3.38
	H	5.81	340	197.54	24.69	9.20	2.66
	I	5.36	318	170.45	21.31	9.20	2.34
	J	6.27	330	206.91	25.86	9.20	2.81
N-7	A	6.90	353	243.57	30.45	9.20	3.24
	B	7.90	356	281.24	35.16	9.20	3.74
	C	7.20	336	241.92	30.24	9.20	3.27
	D	6.90	343	236.67	29.58	9.20	3.18
	E	8.70	338	294.06	36.76	9.20	3.97
N+7	F	14.30	334	477.62	59.70	9.20	6.47
	G	12.60	346	435.96	54.50	9.20	5.84
	H	13.10	332	434.92	54.37	9.20	5.90
	I	11.80	340	401.20	50.15	9.20	5.40
	J	11.20	322	360.64	45.08	9.20	4.94
N-10	A	11.83	331	391.57	48.95	9.20	5.32
	B	10.57	325	343.53	42.94	9.20	4.69
	C	10.13	337	341.38	42.67	9.20	4.61
	D	9.61	316	303.68	37.96	9.20	4.18
	E	9.74	346	337.00	42.13	9.20	4.51
N+10	F	11.31	348	393.59	49.20	9.20	5.26

	G	12.63	340	429.42	53.68	9.20	5.78
	H	12.80	339	433.92	54.24	9.20	5.85
	I	12.18	336	409.25	51.16	9.20	5.53
	J	12.67	349	442.18	55.27	9.20	5.91
P-2	F	4.13	337	139.18	17.40	9.20	1.87
	G	3.81	348	132.59	16.57	9.20	1.78
	H	3.22	353	113.67	14.21	9.20	1.53
	I	2.98	336	100.13	12.52	9.20	1.35
	J	2.85	344	98.04	12.26	9.20	1.32
P+2	A	4.14	368	152.35	19.04	9.20	2.05
	B	4.65	360	167.40	20.93	9.20	2.25
	C	5.21	344	179.22	22.40	9.20	2.41
	D	5.42	343	185.91	23.24	9.20	2.50
	E	5.14	353	181.44	22.68	9.20	2.44
P-4	A	6.04	335	202.34	25.29	9.20	2.72
	B	5.84	334	195.06	24.38	9.20	2.62
	C	5.02	359	180.22	22.53	9.20	2.42
	D	5.47	342	187.07	23.38	9.20	2.51
	E	5.75	345	198.38	24.80	9.20	2.67
P+4	F	7.52	347	260.94	32.62	9.20	3.51
	G	7.40	347	256.78	32.10	9.20	3.45
	H	6.94	337	233.88	29.23	9.20	3.14
	I	6.44	355	228.62	28.58	9.20	3.07
	J	8.32	326	271.23	33.90	9.20	3.65
P-7	A	5.99	348	208.45	26.06	9.20	2.80
	B	5.29	340	179.86	22.48	9.20	2.42
	C	6.17	337	207.93	25.99	9.20	2.79
	D	5.43	349	189.51	23.69	9.20	2.55
	E	5.21	341	177.66	22.21	9.20	2.39
P+7	F	12.91	340	438.94	54.87	9.20	5.90
	G	12.23	348	425.60	53.20	9.20	5.72
	H	13.76	333	458.21	57.28	9.20	6.16
	I	14.56	322	468.83	58.60	9.20	6.30
	J	14.78	326	481.83	60.23	9.20	6.48
P-10	F	6.10	314	191.54	23.94	9.20	2.57
	G	6.63	336	222.77	27.85	9.20	2.99
	H	6.34	338	214.29	26.79	9.20	2.88
	I	7.23	332	240.04	30.00	9.20	3.23
	J	6.73	359	241.61	30.20	9.20	3.25
P+10	F	9.46	346	327.32	40.91	9.20	4.40
	G	10.60	347	367.82	45.98	9.20	4.94

	H	12.11	350	423.85	52.98	9.20	5.70
	I	12.76	356	454.26	56.78	9.20	6.11
	J	14.27	358	510.87	63.86	9.20	6.87

Table S2. Reference table comparing the lipid accumulation of green microalgae species exposed to similar nutrient variations

Species	Lipid % (DCW) range	Lipid % (DCW)	Stress	Lipid response [Reference]
Ettlia oleoabundans	35-54 [1]	29.9	N-	Lipid % (DCW) increased, TAGs and SQDGs accumulated, DGDGs depleted [2]
		58		Lipid % (DCW) and productivity increased [3]
		38		Lipid concentration, productivity and Lipid % (DCW) increased [4]
		35		TAGs accumulated [5]
		38.9		DAGs accumulated over time, TAGs accumulated in exponential growth phase, PAs, PGs, PIs, and PCs depleted, MGMGs, DGDGs, and SQDGs depleted over time [this study]
		19.5	P-	DAGs accumulated over time, TAGs accumulated in P-4 vs P+4, PGs, PIs, and PCs depleted, DGDGs and SQDGs depleted over time [this study]
Chlamydomonas reinhardtii	19-21[6, 7]	NA	N-	TAGs accumulated [8]
		NA		MGDGs, DGDGs, SQDGs and PGs depleted, TAGs accumulated [9]
		NA		saturated TAGs depleted, unsaturated TAGs accumulated [10]
		NA		FAs accumulated over time [11]
		NA		TAGs % (DCW) increased [12]
		NA	P-	TAGs % (DCW) increased [12]
Chlorella sp.	28-32 [1]	50.3	N-	Lipid and TAG productivity increased [13]
		54		Yield of lipids increased [14]
		24.6		Lipid % (DCW) increase [15]
		NA		MGDGs, DGDGs, SQDGs, PEs, and PCs depleted, PGs and TAGs accumulated [16]
		NA		Total FA % (DCW) increased [17]
		46-48		Lipid % (DCW) and productivity increased [3]
		34.3	P-	Lipid productivity increased in the beginning of growth (day 1-4) but not later (day 5-7) [13]
		23.6		Lipid % (DCW) and productivity increased, Neutral lipid and glycolipid increased [18]
Nannochloropsis sp.	31-68 [1]	NA	N-	MGDGs, DGDGs, SQDGs, PEs, and PCs depleted, PGs and TAGs accumulated [16]
		NA		MGDG, DGDG, SQDG, PG, PC, PE, and PI depleted, DAG, TAG and FFA accumulated [19]
			P-	Total lipid accumulation and biomass yield is lower compared to P+ conditions[20]

Table S3. Abundance of lipids analyzed using targeted and untargeted analysis. The *m/z* retention times and ion counts in 16 different sampling conditions are reported (provided as a separate Excel spreadsheet).

References

1. Chisti Y. Biodiesel from microalgae. Biotechnol Adv. 2007;25(3):294-306.
2. Matich EK, Butryn DM, Ghafari M, del Solar V, Camgoz E, Pfeifer BA, Aga DS, Haznedaroglu BZ, Atilla-Gokcumen GE. Mass spectrometry-based metabolomics of value-added biochemicals from Ettlia oleoabundans. Algal Res. 2016;19:146-154.
3. Adams C, Godfrey V, Wahlen B, Seefeldt L, Bugbee B. Understanding precision nitrogen stress to optimize the growth and lipid content tradeoff in oleaginous green microalgae. Bioresour Technol. 2013;131:188-194.
4. Li Y, Horsman M, Wang B, Wu N, Lan CQ. Effects of nitrogen sources on cell growth and lipid accumulation of green alga Neochloris oleoabundans. Appl Microbiol Biotechnol. 2008;81(4):629-636.
5. Rismani-Yazdi H, Haznedaroglu BZ, Hsin C, Peccia J. Transcriptomic analysis of the oleaginous microalga Neochloris oleoabundans reveals metabolic insights into triacylglyceride accumulation. Biotechnol Biofuels. 2012;5(1):74.
6. Li-Beisson Y, Beisson F, Riekhof W. Metabolism of acyl-lipids in Chlamydomonas reinhardtii. Plant J. 2015;82(3):504-522.
7. Scranton MA, Ostrand JT, Fields FJ, Mayfield SP. Chlamydomonas as a model for biofuels and bio-products production. Plant J. 2015;82(3):523-531.
8. Boyle NR, Page MD, Liu B, Blaby IK, Casero D, Kropat J, Cokus SJ, Hong-Hermesdorf A, Shaw J, Karpowicz SJ. Three acyltransferases and nitrogen-responsive regulator are implicated in nitrogen starvation-induced triacylglycerol accumulation in Chlamydomonas. J Biol Chem. 2012;287(19):15811-15825.
9. Siaut M, Cuine S, Cagnon C, Fessler B, Nguyen M, Carrier P, Beyly A, Beisson F, Triantaphylides C, Li-Beisson Y, Peltier G. Oil accumulation in the model green alga Chlamydomonas reinhardtii: characterization, variability between common laboratory strains and relationship with starch reserves. BMC Biotechnology. 2011;11:7.
10. Yang D, Song D, Kind T, Ma Y, Hoefkens J, Fiehn O. Lipidomic Analysis of Chlamydomonas reinhardtii under Nitrogen and Sulfur Deprivation. PLoS One. 2015;10(9):e0137948.
11. Msanne J, Xu D, Konda AR, Casas-Mollano JA, Awada T, Cahoon EB, Cerutti H. Metabolic and gene expression changes triggered by nitrogen deprivation in the photoautotrophically grown microalgae Chlamydomonas reinhardtii and Coccomyxa sp. C-169. Phytochemistry. 2012;75:50-59.
12. Weers PMM, Gulati RD. Growth and reproduction of Daphnia galeata in response to changes in fatty acids, phosphorus, and nitrogen in Chlamydomonas reinhardtii. Limnol Oceanogr. 1997;42(7):1584-1589.
13. Fan J, Cui Y, Wan M, Wang W, Li Y. Lipid accumulation and biosynthesis genes response of the oleaginous Chlorella pyrenoidosa under three nutrition stressors. Biotechnol Biofuels. 2014;7(1):17.
14. Griffiths MJ, van Hille RP, Harrison ST. The effect of degree and timing of nitrogen limitation on lipid productivity in Chlorella vulgaris. Appl Microbiol Biotechnol. 2014;98(13):6147-6159.

15. Mujtaba G, Choi W, Lee C-G, Lee K. Lipid production by Chlorella vulgaris after a shift from nutrient-rich to nitrogen starvation conditions. Bioresour Technol. 2012;123:279-283.
16. Martin GJ, Hill DR, Olmstead IL, Bergamin A, Shears MJ, Dias DA, Kentish SE, Scales PJ, Botte CY, Callahan DL. Lipid profile remodeling in response to nitrogen deprivation in the microalgae Chlorella sp. (Trebouxiophyceae) and Nannochloropsis sp. (Eustigmatophyceae). PLoS One. 2014;9(8)**:**e103389.
17. Zhu SN, Huang W, Xu J, Wang ZM, Xu JL, Yuan ZH. Metabolic changes of starch and lipid triggered by nitrogen starvation in the microalga Chlorella zofingiensis. Bioresour Technol. 2014;152:292-298.
18. Liang K, Zhang Q, Gu M, Cong W. Effect of phosphorus on lipid accumulation in freshwater microalga Chlorella sp. J Appl Phycol. 2013;25(1)**:**311-318.
19. Simionato D, Block MA, La Rocca N, Jouhet J, Marechal E, Finazzi G, Morosinotto T. The Response of Nannochloropsis gaditana to Nitrogen Starvation Includes De Novo Biosynthesis of Triacylglycerols, a Decrease of Chloroplast Galactolipids, and Reorganization of the Photosynthetic Apparatus. Eukaryot Cell. 2013;12(5)**:**665-676.
20. Hu HH, Gao KS. Response of growth and fatty acid compositions of Nannochloropsis sp to environmental factors under elevated CO2 concentration. Biotechnol Lett. 2006;28(13)**:**987-992.
